# Presence of Human Enteric Viruses, Protozoa, and Indicators of Pathogens in the Bagmati River, Nepal

**DOI:** 10.3390/pathogens7020038

**Published:** 2018-04-06

**Authors:** Sarmila Tandukar, Jeevan B. Sherchand, Dinesh Bhandari, Samendra P. Sherchan, Bikash Malla, Rajani Ghaju Shrestha, Eiji Haramoto

**Affiliations:** 1 Department of Natural, Biotic and Social Environment Engineering, University of Yamanashi, 4-3-11 Takeda, Kofu, Yamanashi 400-8511, Japan; sar1234tan@gmail.com (S.T.); mallabikash@hotmail.com (B.M.); rajani_ghaju12@hotmail.com (R.G.S.); 2 Institute of Medicine, Tribhuvan University Teaching Hospital, Kathmandu 44600, Nepal; jeevanbsherchand@gmail.com (J.B.S.); me.dinesh43@gmail.com (D.B.); 3 Department of Global Environmental Health Sciences, Tulane University, 1440 Canal Street, Suite 2100, New Orleans, LA 70112, USA; sshercha@tulane.edu; 4 Interdisciplinary Center for River Basin Environment, University of Yamanashi, 4-3-11 Takeda, Kofu, Yamanashi 400-8511, Japan

**Keywords:** Bagmati River, enteric virus, human-fecal marker, index virus, protozoa

## Abstract

Quantification of waterborne pathogens in water sources is essential for alerting the community about health hazards. This study determined the presence of human enteric viruses and protozoa in the Bagmati River, Nepal, and detected fecal indicator bacteria (total coliforms, *Escherichia coli*, and *Enterococcus* spp.), human-fecal markers (human *Bacteroidales* and JC and BK polyomaviruses), and index viruses (tobacco mosaic virus and pepper mild mottle virus). During a one-year period between October 2015 and September 2016, a total of 18 surface water samples were collected periodically from three sites along the river. Using quantitative polymerase chain reaction, all eight types of human enteric viruses tested—including adenoviruses, noroviruses, and enteroviruses, were detected frequently at the midstream and downstream sites, with concentrations of 4.4–8.3 log copies/L. Enteroviruses and saliviruses were the most frequently detected enteric viruses, which were present in 72% (13/18) of the tested samples. *Giardia* spp. were detected by fluorescence microscopy in 78% (14/18) of the samples, with a lower detection ratio at the upstream site. *Cryptosporidium* spp. were detected only at the midstream and downstream sites, with a positive ratio of 39% (7/18). The high concentrations of enteric viruses suggest that the midstream and downstream regions are heavily contaminated with human feces and that there are alarming possibilities of waterborne diseases. The concentrations of enteric viruses were significantly higher in the dry season than the wet season (*p* < 0.05). There was a significant positive correlation between the concentrations of human enteric viruses and the tested indicators for the presence of pathogens (IPP) (*p* < 0.05), suggesting that these IPP can be used to estimate the presence of enteric viruses in the Bagmati River water.

## 1. Introduction

The Bagmati River, an important water resource of Nepal, is currently facing biological, chemical, and other ecological challenges [1,2,3]. Due to an inadequate water supply, untreated water from this river is used by many inhabitants [4,5] for various purposes, including for irrigation [6], cleaning of freshly harvested crops products [7], and domestic use [8]. Moreover, uncontrolled population growth and unplanned urbanization have led to most sewage and solid waste from urban areas directly discharging into rivers [9]. Therefore, the presence of diverse groups of waterborne pathogens in this river water is likely high [10]. The characterization and quantification of waterborne pathogens in the Bagmati River are essential to identify the sources and potential risks from contamination.

The lack of molecular diagnostic laboratories in Nepal means that human enteric viruses are not commonly diagnosed from environmental and clinical samples at the national level. Although several studies have assessed the water quality of the Bagmati River [1,2,3,4,7,8,9,11,12], few have analyzed the microbiological aspect of water quality with respect to the presence of enteric viruses, protozoa, and bacteria [7,12,13,14,15,16]. The available data regarding pathogen contamination in the Bagmati River and its impacts on health and the community are limited. Thus, more studies are needed to address this knowledge gap.

Although diverse groups of waterborne pathogens are present in river water [17], the lack of standardized techniques and high costs mean it is almost impossible to detect all the waterborne pathogens simultaneously [18]. Consequently, routine monitoring of fecal indicator bacteria is traditionally used to provide correlative information on human enteric viruses and protozoa. However, the fecal indicator bacteria method has a low correlation with the presence of pathogens, and it cannot be used to identify sources of contamination [19]. Additionally, *Escherichia coli*, *Enterococcus* spp., and other fecal indicator bacteria are shed in the feces of many different animals [20,21]: thus making it further challenging to identify the actual sources of contamination simply by measuring these indicators.

Microbial source tracking using host-specific genetic markers of *Bacteroidales* is a promising library-independent method used to differentiate between human and animal fecal-source contamination [22,23]. HF183 is one of the most famous *Bacteroidales* markers used to evaluate uniform distribution and potential sources of human sewage contamination [21]. Some enteric viruses, such as adenoviruses [24] and polyomaviruses [25], are listed as index viruses as well as markers of human wastewater, which are used to estimate the concentration of pathogenic viruses in different water sources. Pepper mild mottle virus (PMMoV) and tobacco mosaic virus (TMV) have been proposed as potential fecal indicators in water due to their abundant presence in the human stool and sewage samples [14]. The fate and transportation of enteric viruses and protozoa in the environment and during water treatment processes can differ from those of fecal indicator bacteria [26]. Therefore, it is essential to develop reliable and applicable fecal indicator bacteria, human-fecal markers, and index viruses to obtain appropriate correlations with enteric viruses.

To the best of our knowledge, this study is the first to determine the presence of human enteric viruses and protozoa in the Bagmati River using samples obtained along the major settlement zone of the Kathmandu Valley. A total of 18 river water samples collected at three sites along the Bagmati River during a one-year period were examined for the presence of eight human enteric viruses and two protozoa using (reverse transcription-)quantitative polymerase chain reaction ((RT-)qPCR) and fluorescence microscopy, respectively. This study also assessed fecal indicator bacteria, human-fecal markers, and index viruses to identify reliable indicators for the presence of pathogens (IPP) in river water.

## 2. Results

### 2.1. Detection of Fecal Indicator Bacteria, Human-Fecal Markers, and Index Viruses

Table 1 provides a summary of the results of the human enteric viruses, protozoa, and indicators detected in the river water samples. Total coliforms and *E. coli* were detected in all tested samples, whereas *Enterococcus* spp. were detected in 94% (17/18) of the samples. TMV and PMMoV were identified using RT-qPCR and were detected in 89% (16/18) and 78% (14/18) of the tested samples, respectively. Among the three human-fecal markers tested, BK and JC polyomaviruses (BKPyVs and JCPyVs) were detected in 56% (10/18) and 50% (9/18) of the tested samples, respectively, whereas human *Bacteroidales* were detected in 89% (16/18) of the samples.

### 2.2. Detection of Protozoa

**Figure 1:**
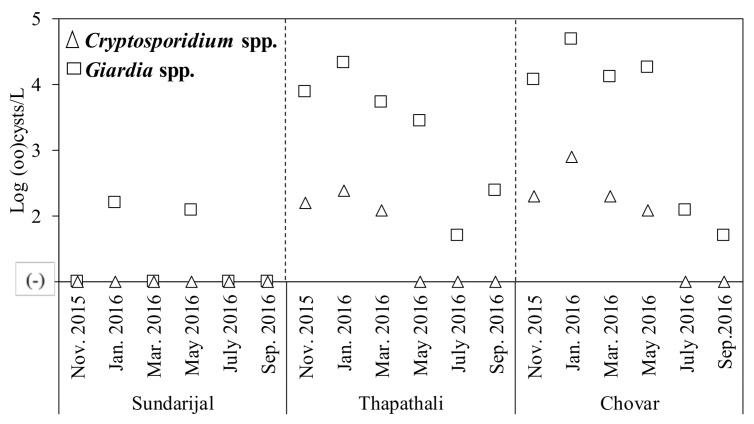
Concentration of protozoa in the river water samples over time.

Table 2 provides a summary of the results of the protozoa and enteric viruses detected in the river water samples. Based on fluorescence microscopy, *Cryptosporidium* spp. and *Giardia* spp. were detected in 39% (7/18) and 78% (14/18) of the tested samples, respectively. At the upstream site (Sundarijal), *Cryptosporidium* spp. were not detected in any of the tested samples, and *Giardia* spp. were detected only in two (33%) of the tested samples. By contrast, at the midstream (Thapathali) and downstream sites (Chovar), *Giardia* spp. were detected in all tested samples, with high concentrations of 1.6–4.7 log cysts/L (Figure 1). Similarly, *Cryptosporidium* spp. were detected in 7 out of 12 (58%) samples collected at Thapathali and Chovar, with concentrations of 2.1–2.9 log oocysts/L (Figure 1).

### 2.3. Detection of Human Enteric Viruses

Eight human enteric viruses (Aichi virus 1 (AiV-1), enteroviruses (EVs), human cosaviruses (HCoSVs), human adenoviruses (HuAdVs), noroviruses of genogroups I (NoVs-GI) and II (NoVs-GII), group A rotaviruses (RVAs), and saliviruses (SaliVs)) were analyzed at three different sites along the Bagmati River (Table 2). Among them, only half the human enteric viruses (AiV-1, EVs, NoVs-GII, and SaliVs) were detected in the samples collected at Sundarijal. By contrast, all human enteric viruses tested were frequently detected at Thapathali and Chovar, with concentrations of 4.4–8.3 log copies/L. Of all the tested samples, EVs and SaliVs were the most prevalent human enteric viruses, with a positive ratio of 72% (13/18), followed by AiV-1 and HuAdVs (67%, 12/18).

**Figure 2:**
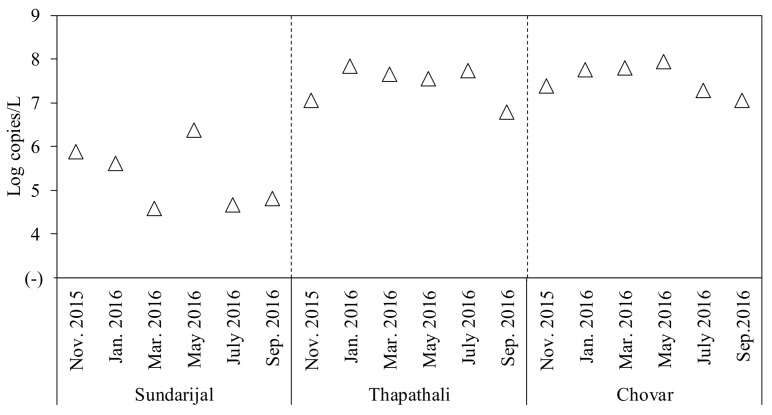
Concentrations of total human enteric viruses in the river water samples.

The highest concentrations of AiV-1 (6.6 log copies/L), EVs (7.5 log copies/L), and SaliVs (8.3 log copies/L) were observed at Chovar, whereas HCoSVs (6.7 log copies/L) and NoVs-GII (5.7 log copies/L) were detected with the highest concentrations at Thapathali in March 2016. The concentration of HuAdVs (7.7 copies/L) was highest at Thapathali in July 2016. As shown in Figure 2, the concentrations of total human enteric viruses, which were calculated as an arithmetic sum of the concentrations of eight human enteric viruses, were higher at Chovar and Thapathali than at Sundarijal (*p* < 0.05). For Thapathali and Chovar, increased concentrations of total human enteric viruses were observed between January and May 2016 (*p* < 0.05).

### 2.4. Relationships Between Pathogens and Indicators

Table 3 provides a summary of the results of statistical analyses to determine relationships between pathogens (protozoa and total human enteric viruses) and indicators (fecal indicator bacteria, human-fecal markers, and index viruses) in the river water samples. Total human enteric viruses showed a significant positive correlation with these indicators (correlation coefficient (*R*), 0.49–0.81; *p* < 0.05). By contrast, there was no positive correlation for any of the three fecal indicator bacteria and *Cryptosporidium* spp. and *Giardia* spp. (*p* > 0.05). For *E. coli*, similar results were obtained between qPCR (Table 3) and the Colilert methods (IDEXX Laboratories, Westbrook, CA, USA) (data not shown).

## 3. Discussion

Our study confirmed the presence of human enteric viruses and protozoa in water obtained from the Bagmati River, which is used potentially for domestic and recreational activities. High concentrations of these pathogens in the Bagmati River water are a serious threat for the people who live in its proximity. For instance, splash and aerosols from the river might be inhaled or ingested by people, leading to acute or chronic infections particularly in immunocompromised patients, pregnant women, children, and elderly people.

Previous studies of water samples obtained from the Kathmandu Valley showed that viruses—such as AiV-1 [13], SaliVs, EVs, HuAdVs [14], NoVs-GI, and NoVs-GII [12]—were the predominant contaminants, which our results confirm. In this study, human enteric viruses were frequently detected in the tested samples, except for those collected at Sundarijal, where only four of the eight virus types tested were detected and at relatively low concentrations. Human enteric viruses are the major causative agents of diarrhea in children and more often in immunocompromised patients. However, data about causative agents of diarrheal diseases due to human enteric viruses in Nepal are scarce. Only limited studies have been conducted previously for these human enteric viruses from diarrheal stool [27,28,29,30,31,32]. The high occurrence of SaliVs, EVs, HuAdVs, and AiV-1 in river water indicates the existence of these human enteric viruses in the community [33], although they have been less commonly or not diagnosed in clinical laboratories. Therefore, more studies should be conducted on sewage and diarrheal samples to obtain reliable conclusions regarding river water contamination.

The abundance of *Giardia* spp. and *Cryptosporidium* spp. in the river water samples was in accordance with previous studies [7,12]. However, these studies were based on a single-time sampling, whereas this study collected samples over one year. The analysis of the seasonal trend of protozoa revealed that positive ratios of *Giardia* spp. and *Cryptosporidium* spp. were higher in the dry season than in the wet season, although this difference was not significant (*p* > 0.05). *Giardia duodenalis* is a major cause of diarrhea in Nepal [34], and *Cryptosporidium* spp. in particular cause diarrhea in immunocompromised patients [35]. These intestinal protozoa were detected from clinical samples during almost entire season in the Kathmandu Valley [36].

The chemical, microbiological, and physical parameters at Sundarijal were within acceptable levels according to the Bagmati Basin Water Management Strategy and Investment Program guideline [8,9]. A previous study conducted in this region reported that these upstream sites of the river are minimally affected by anthropogenic activities [37]. However, as the river passes through settlement areas, it becomes contaminated. Therefore, the degradation of the river water quality as it passes downstream is likely due to human activities. Indeed, the midstream and downstream sites were heavily polluted with bacteria, protozoa, and viruses. Even though the detection ratio of pathogens from the upstream site was low, potential contamination cannot be neglected.

There was a significant positive correlation between total human enteric viruses and IPP. The detection ratios and the concentrations of TMV and PMMoV were relatively higher than those of the human enteric viruses; this result is comparable with those of a previous study that studied the virological quality of irrigation water sources in the Kathmandu Valley [14]. This association showed that these index viruses could be a reliable tool for monitoring the presence of human enteric viruses in river water samples.

However, there was no significant correlation between fecal indicator bacteria and protozoa. This lack of correlation might be due to the widely different physiology and phylogeny between fecal indicator bacteria and protozoa [21]. In this study, the detection ratio and the concentrations of human *Bacteroidales* were higher than other human-fecal markers, with a significant correlation with human enteric viruses. The bacterial indicators, index viruses, and human *Bacteroidales* could be a reliable indicator of human-fecal contamination in river water, and a bacterial indicator is still a good signal for the presence of human enteric viruses. By contrast, the IPP were not sufficient to indicate the presence of protozoa (*p* > 0.05); therefore, further studies are required to develop reliable IPP of protozoal contamination.

Only a small number of samples from three different sampling sites were analyzed in this study, which might not be sufficient to provide a complete picture of the level of contamination in the Bagmati River. Nevertheless, we suggest that non-human sources of contamination must be identified and measured because these data are essential for tracing the sources of contamination. Indeed, human pathogens—such as intestinal parasites and bacteria—are often present in animal fecal matter and represent high risks to health; thus, it is essential to know the level of risk associated with animal fecal material [38].

In conclusion, this study successfully demonstrated that the Bagmati River is contaminated with various types of human enteric viruses and protozoa, with alarming risks of diarrhea to the surrounding communities. The more abundant occurrence of human-fecal markers in the midstream and downstream sites than the upstream site indicated that the Bagmati River is extremely degraded by human activities. Our survey suggested that the index viruses, human-fecal markers, and fecal indicator bacteria could be reliable indicators for the presence of human enteric viruses.

## 4. Materials and Methods

### 4.1. Collection of River Water Samples

The sampling sites selected consisted of the upstream region of the Bagmati River (Sundarijal), the midway region as the river passes through the settlement area of the highest population density (Thapathali), and the downstream region of the least population density (Chovar) as the river flows beyond the town limits. Water samples were collected in 100-mL sterile, plastic bottles every 2 months during a 12-month period (November 2015 and September 2016). All samples were transported to the laboratory in dry ice, kept at 4 °C, and processed within 4 h.

### 4.2. Detection of Total Coliforms and E. coli

Total coliforms and *E. coli* in the samples were determined by the most probable number (MPN) method using a Colilert reagent according to the manufacturer’s protocol. After incubation at 37 °C for 24 h, yellow wells were counted as total coliforms, whereas fluorescent blue wells under exposure to UV light were regarded as *E. coli*. Both the small and large wells were counted, and the MPN value for the *E. coli* and total coliforms present in 100-mL of the water samples were determined using an MPN generating software (IDEXX Laboratories).

### 4.3. Detection of Bacteria Using qPCR

A sterilized disposable filter unit with a nitrocellulose membrane (pore size, 0.22-μm; diameter, 47-mm; Nalgene, Tokyo, Japan) was used to concentrate 10-mL of the river water sample, and bacterial DNA was extracted using CicaGeneus DNA Extraction Reagent (Kanto Chemical, Tokyo, Japan), as described previously [16]. Hydrolysis probe-based qPCR targeting an 83 bp fragment of the *uidA* gene of *E. coli* [39] and targeting a 90 bp fragment of 23S rRNA gene of *Enterococcus* spp. [40] were carried out. Similarly, SYBR Green-based qPCR assays were performed for a human-specific *Bacteroidales* marker (HF183), targeting an 82 bp fragment of the 16S rRNA gene [41]. All qPCR assays were performed using a StepOne Real-Time PCR System (Applied Biosystems, New York, NY, USA). For hydrolysis probe-based qPCR, 20-µL of a reaction mixture containing 10-µL of 2 × PerfeCTa qPCR ToughMix (Quanta Biosciences, Beverly, MA, USA), 0.5-µM each of forward and reverse primers, 0.5-µM of a TaqMan probe (Integrated DNA Technologies, Coralville, IA, USA), and 2.5-µL of the template DNA was prepared. The qPCR reactions for *E. coli* and *Enterococcus* spp. were performed in duplicate, and amplification protocols were as follows: 95 °C for 3 min, followed by 45 cycles of 95 °C for 10 s, 60 °C for 10 s, and 72 °C for 10 s for *E. coli*, and 95 °C for 2 min, followed by 40 cycles of 95 °C for 15 s and 60 °C for 60 s for *Enterococcus* spp. SYBR Green-based qPCR reaction mixture (15-µL) contained 7.5-µL of SsoAdvanced Universal SYBR Green Supermix (Bio-Rad, Hercules, CA, USA), 0.25-µM each of forward and reverse primers, and 2.5-µL of the template DNA, and the qPCR reactions were performed in duplicate. The amplification conditions were 95 °C for 10 min, followed by 40 cycles of 95 °C for 15 s, 60 °C for 30 s, and 72 °C for 30 s.

Quantitative DNA standards were obtained from American Type Culture Collection (ATCC, Manassas, VA, USA) using commercial genomic DNA from *E. coli* (ATCC 700926DQ) and *Enterococcus* spp. (ATCC 29212Q-FZ). For the SYBR Green-based qPCR assay, plasmid DNA was obtained from Dr. Min Feng (Auburn University, Auburn, AL, USA). A calibration curve was prepared using fold serial dilutions of the standard samples, and no-template controls were included in each run. The qPCR reactions were performed in duplicate, and the cycle threshold (Ct) value was determined using StepOne Real-Time PCR System Software (Applied Biosystems).

### 4.4. Virus and Protozoan Concentration Methods

Viruses and protozoa in the river water sample were concentrated using the electronegative membrane-vortex method [42]. Briefly, 500-µL of 2.5 M MgCl_2_ was added to 50-mL of the sample, which was then passed through a mixed cellulose ester membrane (pore size, 0.8-μm; diameter, 47-mm; Merck Millipore, Billerica, MA, USA) attached to a plastic filter holder (Advantec, Tokyo, Japan) using a vacuum pump system. The membrane was subsequently removed from the filter holder, followed by vigorous vortexing of the membrane with 10-mL of elution buffer containing 0.2-g/L Na_4_P_2_O_7_ 10H_2_O, 0.3 g/L C_10_H_13_N_2_O_8_Na_3_ 3H_2_O, and 0.1-mL/L Tween 80 in a 50-mL plastic tube. A similar procedure was repeated using 5-mL of elution buffer, which was transferred into the same plastic tube. The sample was centrifuged at 2000× *g* for 10 min at 4 °C, and the resulting pellet was used for protozoa analysis, whereas supernatant was used for virus analysis. The supernatant was further concentrated using a Centriprep YM-50 ultrafiltration device (Merck Millipore) according to the manufacturer’s protocol.

### 4.5. Detection of Cryptosporidium and Giardia (Oo)Cysts Using Fluorescence Microscopy

The pellet obtained after centrifugation step was suspended in 10-mL of phosphate-buffered saline (PBS (−)) and centrifuged again at 2000× *g* for 10 min at 4 °C. The resulting pellet was resuspended in 10-mL of PBS (−) after removing the supernatant. To purify *Cryptosporidium* and *Giardia* (oo)cysts, the sample was subjected to immunomagnetic separation (IMS) using a Dynabeads GC-Combo (Life Technologies, Carlsbad, CA, USA) as described previously [43]. Subsequently, half of the IMS-purified sample (110-μL) was passed through a hydrophilic polytetrafluoroethylene membrane (pore size, 1.0-μm; diameter, 25-mm; Advantec), followed by fluorescence staining of the protozoan (oo)cysts on the membrane using an EasyStain (BTF, North Ryde, Australia). A BX53 fluorescence microscope (Olympus, Tokyo, Japan) was used to count the numbers of *Cryptosporidium* oocysts and *Giardia* cysts.

### 4.6. Detection of Viral DNA Using qPCR

Two hundred microliters of the virus-concentrated sample was subjected to viral DNA extraction using a QIAamp DNA Mini Kit (QIAGEN, Hilden, Germany) and a QIAcube instrument (QIAGEN). Briefly, 2.5-µL of the extracted viral DNA (200-µL) was mixed with 22.5-µL of a PCR mixture containing 12.5-µL of Probe qPCR Mix (Takara Bio, Kusatsu, Japan), 0.4-µM each of forward and reverse primers, and 0.2-µM of a TaqMan based probe (Thermo Fisher Scientific, Waltham, MA, USA). Subsequently, PCR tubes containing the mixtures were placed in a Thermal Cycler Dice Real Time System TP800 (Takara Bio) and incubated at 95 °C for 30 s, followed by 45 cycles of 95 °C for 5 s and 58 °C (for HuAdVs) or 60 °C (for BKPyVs and JCPyVs) for 30 s. Artificially synthesized plasmid DNA containing the target viral sequence was used as a standard sample. The concentration of each virus was calculated based on the standard curve prepared using tenfold serial dilutions of the standard samples, and samples were considered negative if the Ct value was greater than 40; subsequently, the Ct value was determined using Thermal Cycler Dice Real Time System Software (Takara Bio). The slope of the standard curve was used to calculate a PCR amplification efficiency. The data were collected from runs with standard curves that had *R*^2^ values of ≥0.96 and amplification efficiencies of 96–120%.

### 4.7. Detection of Viral RNA Using RT-qPCR

One microliter of a stock solution of MNV (S7-PP3 strain), kindly provided by Dr. Yukinobu Tohya (Nihon University, Fujisawa, Japan), was inoculated into 140-µL of the virus-concentrated water sample as a MPC as recommended elsewhere [44]. The MNV-MPC was also added to 140-µL of PCR-grade water to prepare a non-inhibition control sample, which is essential to calculate the efficiency of extraction- RT-qPCR. The MNV-MPC-inoculated sample was subjected to viral RNA extraction using a QIAamp Viral RNA Mini Kit (QIAGEN) and a QIAcube instrument to obtain a final volume of 60-µL. Thirty-five microliters of the extracted RNA was subjected to RT using a High-Capacity cDNA Reverse Transcription Kit (Thermo Fisher Scientific) to synthesize 70-µL of cDNA.

TaqMan (MGB)-based qPCR was performed for seven human enteric viruses (AiV-1 [45], EVs [46,47], HCoSVs [48], NoVs-GI and NoVs-GII [49], RVAs [50], and SaliVs [51]), two index viruses (PMMoV [52,53] and TMV [54]), and MNV [55]. qPCR for these viruses was performed similarly with DNA viruses, with the following thermal conditions: 95 °C for 30 s, followed by 45 cycles of 95 °C for 5 s and 58 °C for 30 s (for AiV-1, NoVs-GI, NoVs-GII, and SaliVs), 58 °C for 60 s (for HCoSVs), or 60 °C for 60 s (for PMMoV, TMV, EVs, RVAs, and MNV). The standard curves were used for data analysis, with *R*^2^ values of ≥0.96 and amplification efficiencies of 96–120%. The mean efficiency of MNV-MPC was calculated to be 51 ± 57% (*n* = 18), suggesting that there was no significant loss during the detection processes tested.

### 4.8. Statistical Analysis

The concentrations of the viruses, protozoa, and bacteria were expressed as log_10_ values. The total concentration of human enteric viruses was calculated as an arithmetic sum of the concentrations of eight human enteric viruses tested. October to May was considered as a dry season, whereas June to September (pre-monsoon) was considered as a wet (monsoon) season. The rainfall is more intense during the monsoon season, with 65% of the annual total rainfall [56]. The statistical analysis was performed using IBM SPSS Statistics Version 20.0 (IBM Corporation, Armonk, NY, USA). The relationships between the total concentrations of human enteric viruses and those of indicators (fecal indicator bacteria, human-fecal markers, and index viruses) were determined by calculating bivariate correlation with Pearson coefficients, where *p* < 0.05 was considered significant. McNemar’s test was performed to evaluate the seasonal variation in the positive ratios of protozoa by comparing paired groups of nominal data, whereas Wilcoxon test was performed to determine the variation in concentrations of tested pathogens (protozoa and human enteric viruses) among the three different sampling sites and in two different seasons. For the negative samples, one-tenth of limit of detection values (4.2–5.5 log copies/L for DNA viruses, 3.0–4.3 log copies/L for RNA viruses, 1.6 log (oo)cysts/L for protozoa, and 2.5 log copies/L for *Enterococcus* spp.) were used for the statistical analysis.

## Figures and Tables

**Table 1:** Positive ratios of indicator bacteria, potential index viruses, and human-fecal markers in the river water samples

Types	Microorganisms Tested	No. of Positive Samples (%)				Concentrations Among Positive Samples	
		Sundarijal (*n =* 6)	Thapathali (*n =* 6)	Chovar (*n =* 6)	Total (*n =* 18)	Range (min–max)	Unit
Indicator bacteria	Total coliforms	6 (100)	6 (100)	6 (100)	18 (100)	4.8–10.3	Log MPN/100-mL
	*E. coli*	6 (100)	6 (100)	6 (100)	18 (100)	3.5–10.0 6.2–8.6	Log MPN/100-mL Log copies/L
	*Enterococcus* spp.	5 (83)	6 (100)	6 (100)	17 (94)	5.6–10.1	Log copies/L
Potential index viruses	TMV	5 (83)	6 (100)	5 (83)	16 (89)	5.1–9.0	Log copies/L
	PMMoV	3 (50)	6 (100)	5 (83)	14 (78)	4.4–8.0	Log copies/L
Human-fecal markers	BKPyVs	0 (0)	5 (83)	5 (83)	10 (56)	6.0–7.1	Log copies/L
	JCPyVs	4 (67)	5 (83)	0 (0)	9 (50)	6.0–7.0	Log copies/L
	Human *Bacteroidales*	4 (67)	6 (100)	6 (100)	16 (89)	6.5–9.8	Log copies/L

**Table 2:** Positive ratios of protozoa and human enteric viruses in the river water samples

Types	Microorganisms Tested	No. of Positive Samples (%)				Concentrations Among Positive Samples	
		Sundarijal (*n =* 6)	Thapathali (*n =* 6)	Chovar (*n =* 6)	Total (*n =* 18)	Range (min–max)	Unit
Protozoa	*Cryptosporidium* spp.	0 (0)	3 (50)	4 (67)	7 (39)	2.1–2.9	Log oocysts/L
	*Giardia* spp.	2 (33)	6 (100)	6 (100)	14 (78)	1.6–4.7	Log cysts/L
Human enteric viruses	AiV-1	1 (17)	6 (100)	5 (83)	12 (67)	4.6–6.6	Log copies/L
	EVs	2 (33)	6 (100)	5 (83)	13 (72)	5.4–7.5	Log copies/L
	HCoSVs	0 (0)	5 (83)	4 (67)	9 (50)	5.7–6.7	Log copies/L
	HuAdVs	0 (0)	6 (100)	6 (100)	12 (67)	6.2–7.7	Log copies/L
	NoVs-GI	0 (0)	4 (67)	1 (17)	5 (28)	4.4–5.0	Log copies/L
	NoVs-GII	2 (33)	6 (100)	3(33)	11 (61)	4.9–5.7	Log copies/L
	RVAs	0 (0)	1 (17)	2 (33)	3 (17)	4.5–5.0	Log copies/L
	SaliVs	2 (33)	6 (100)	5 (83)	13 (72)	4.4–8.3	Log copies/L

**Table 3:** Relationships in the concentrations between pathogens and indicators

Types	Indicators	*R* Value		
		Total Human Enteric Viruses	*Cryptosporidium* spp.	*Giardia* spp.
Fecal indicator bacteria	Total coliforms	0.56 *	0.20	0.14
	*E. coli*	0.69 *	0.24	0.17
	*Enterococcus* spp.	0.73 *	0.30	0.30
Human-fecal markers	BKPyVs	0.81 *	0.06	0.13
	JCPyVs	0.74 *	0.09	0.01
	Human *Bacteroidales*	0.71 *	0.11	0.09
Index viruses	TMV	0.49 *	0.01	0.13
	PMMoV	0.76 *	0.12	0.21

* Statistically significant (*p* < 0.05).
